# “Inconsistency between words and deeds”: a meta-analysis of the moderating and mediating mechanisms of bridging the exercise-intentional-behavior gap

**DOI:** 10.3389/fpsyg.2025.1586176

**Published:** 2025-05-21

**Authors:** Zexin Yang, Jun Li, Xiaoming Liu

**Affiliations:** ^1^School of Physical Education, Hunan University of Technology, Zhuzhou, China; ^2^School of Physical Education, Hunan Normal University, Changsha, Hunan, China

**Keywords:** exercise intention, exercise behavior, intention-behavior gap, meta-analysis, BCT

## Abstract

Exercise intention is a crucial predictor of exercise behavior; however, the existence of the “intention-behavior gap” is an undeniable fact, and the strength and mechanisms of their relationship remain controversial. This study, based on 92 empirical studies (109 independent samples, 47,548 participants), employs meta-analysis to examine the relationship strength between exercise intention and behavior, the moderating effects of participant characteristics, and the mediating role of planning and action control. The findings are as follows: (1) the relationship strength between exercise intention and behavior is moderate (*r* = 0.41); (2) the moderating effects of age, educational stage, health status, socioeconomic status, cultural background, and economic level are significant, except for gender; (3) action planning, coping planning, and action control mediate the relationship between exercise intention and behavior in a chain-like manner, with action control (Single mediation effect was 13.45%) being the closest predictor of behavior. This undoubtedly provides inspiration for the formulation of intervention strategies. Strengthening action control as the core target, supplemented by action plans and coping plans can better promote the implementation of exercise behavior. Future research is recommended to strictly control participant characteristics, conduct long-term longitudinal tracking and experimental interventions, strengthen the use of objective measurement tools, and explore and analyze new theories and variables to facilitate the translation of exercise intention into behavior.

## Introduction

Physical exercise is crucial for the development of both physical and mental health. Regular physical activity not only aids in the prevention and management of non-communicable diseases but also alleviates mental health issues and enhances overall wellbeing. Despite widespread recognition of the benefits of exercise, 28% of adults and 81% of adolescents worldwide still fail to meet recommended physical activity levels (World Health Organization, 2024). To encourage participation, traditional social-cognitive models, such as the Theory of Planned Behavior (TPB), regard intention as the proximal determinant of behavior (Conner and Norman, 2015). It is evident that the formation of intention is conducive to behavioral change (McEachan et al., 2011), yet scholars have discovered that individuals often encounter numerous barriers—stemming from work, study, and environmental factors—that hinder the translation of exercise intentions into actual behavior, creating a “gap between intention and action” (Rhodes and de Bruijn, 2013; Rhodes et al., 2012; Feng and Mao, 2014). Furthermore, research based on the Health Action Process Approach (HAPA) theory has demonstrated that a staggering 82% of the variance in exercise behavior remains unexplained by intention alone (Liu X. M. et al., 2022), indicating that additional variables are essential to better understand behavioral change. Although Feil et al. (2023) quantified this disparity through a meta-analysis based on the action control framework, the research focused on individual psychological mechanisms (such as self-efficacy, etc.), while neglecting the moderating roles of the environment (such as community facilities and policy support, etc.) and socioeconomic status (such as income and education, etc.) in the transformation from intention to behavior. For example, individuals in resource-deficient regions may have greater difficulty in realizing their intentions due to structural obstacles. However, such factors were not incorporated into the moderating analysis, resulting in the design of intervention strategies being overly reliant on the individual level and disregarding the need for systemic support.

As research proliferates, how significant is the impact of exercise intention on exercise behavior (main effect)? What factors influence the relationship between the two (moderating mechanisms)? How does exercise intention mediate the connection to behavior (mediating mechanisms)? Scholars continue to hold differing views, and no consensus has been reached. Exploring the relationship between exercise intention and behavior, along with the underlying mechanisms, requires not only an understanding of the strength of their connection but also an uncovering of the potential, multifaceted moderating and mediating variables at play. Furthermore, how might individual differences and environmental factors act as “catalysts” or “inhibitors” that influence the strength of the relationship between intention and behavior? How do they construct the bridge from intention to action? Unraveling these mysteries will help to formulate precise intervention strategies and optimize the path of transformation from intention to behavior, thereby promoting greater participation in exercise and enhancing physical and mental wellbeing.

Therefore, this study aims to employ a meta-analytic approach to compare the similarities and differences across previous research, exploring the strength of the relationship between exercise intention and behavior, the moderating effects of participant characteristics, and the mediating role of planning and action control. This not only serves to minimize or eliminate measurement and sampling errors inherent in individual studies, thereby enhancing the external validity of the findings (Hunter and Schmidt, 2004), but also allows for the examination of the relationship strength and underlying mechanisms between exercise intention and behavior. Furthermore, by identifying various moderating and mediating variables, it aims to develop personalized exercise promotion strategies tailored to individuals from different social backgrounds.

### The relationship between exercise intention and behavior

Exercise intention refers to the extent to which an individual subjectively desires to engage in physical activity (Yang, 2012). The effect of exercise intention on exercise behavior has been confirmed by a large number of studies (Fang, 2012; Yang et al., 2020). However, experimental interventions aimed at enhancing exercise intention (*d* = 0.45) have not resulted in the anticipated increase in exercise behavior (*d* = 0.15; Rhodes et al., 2012). This suggests that the relationship between intention and behavior remains uncertain. In any field of study, the outcomes of research are influenced by the personalized design and sample estimations of the researcher, which may introduce biases when compared to the broader context (Jin et al., 2023). Therefore, the discrepancies in past empirical studies on the relationship between exercise intention and behavior may stem from differences in sample characteristics and research attributes, such as measurement tools and types of exercise. Specifically, sample characteristics encompass individual differences and environmental factors. On an individual level, these include age, gender, educational background, socioeconomic status, health status, motivation, and self-efficacy; on an environmental level, they include access to exercise facilities, weather, environment, social support, national economic status, and cultural background. Thus, it is essential to use meta-analysis to integrate research on the relationship between exercise intention and behavior to provide further evidence of the strength of their connection, while reducing biases that may arise from individual differences and varying socio-cultural contexts.

### The moderating mechanism of participant characteristics in the relationship between exercise intention and behavior

Meta-analyses typically derive potential moderating variables by coding empirical literature, which are usually categorized into two types: one being measurement factors, such as measurement methods and dimensional divisions; the other being contextual factors, such as participant characteristics (Liu and Qin, 2018). Research has shown that the conversion of exercise intentions into behavior is influenced by various factors (Rhodes et al., 2022). The effect of measurement factors has been confirmed (Feil et al., 2023), yet there has been limited scholarly focus on contextual factors. Therefore, this study exclusively analyzes the moderating effects of contextual factors (i.e., participant characteristics).

Through a review of the literature, it was found that the research results of different groups of participants with varying characteristics exhibit considerable discrepancies. For example, the strength of the relationship between exercise intention and behavior in adolescents was as high as *r* = 0.7 and *r* = 0.77 (Downs et al., 2006; Hagger et al., 2007), whereas in older adults, the strength of this relationship was only *r* = 0.1 and *r* = 0.08 (Blanchard et al., 2009; Sniehotta et al., 2010), suggesting that age may influence the strength of the relationship. Additionally, the strength of the relationship between exercise intention and behavior in females and males was *r* = 0.61 and *r* = 0.19, respectively (Fleig et al., 2013; Schwarzer et al., 2008), indicating a potential gender effect. For participants with suboptimal health and those with good health, the relationship strength between intention and behavior was *r* = 0.69 and *r* = 0.77, respectively, implying that health status may not significantly affect this relationship (Gao et al., 2024; Hagger et al., 2007). Among secondary school and university students, the relationship strength between exercise intention and behavior was *r* = 0.16 and *r* = 0.7, respectively, suggesting that the relationship may be influenced by educational level (Conner et al., 2010; Hashim et al., 2014). Moreover, varying socioeconomic status may also impact the strength of this relationship, as demonstrated by the exercise intention-behavior relationship strengths of *r* = 0.67 and *r* = 0.27 for middle and lower socioeconomic status groups, respectively (Downs and Hausenblas, 2003; Johnson et al., 2015). Cross-cultural studies reveal that the relationship between exercise intention and behavior may vary depending on national cultural backgrounds and economic development levels. For instance, research by Wang and MacCann, conducted in Eastern and Western cultural contexts, found relationship strengths of *r* = 0.14 and *r* = 0.59, respectively (MacCann et al., 2015; Wang and Zhang, 2016). Similarly, studies by Lee and Hu in developed and developing countries revealed relationship strengths of *r* = 0.48 and *r* = 0.036 (Hu et al., 2023; Lee and Lee, 2020).

To sum up, under the circumstances of different ages, genders, health conditions, educational stages, socioeconomic statuses, and the cultural and economic backgrounds of the countries where the subjects are located, the research findings are quite disparate. Therefore, recognizing the significance of different variables in the process of converting intention into behavior under different sample characteristics is of paramount importance for theoretical development and practical application. Hence, this study intends to adopt the method of meta-analysis to comprehensively evaluate whether age, gender, health condition, educational stage, socioeconomic status, cultural background, and economic level will influence the relationship between exercise intention and behavior, aiming to deepen the understanding of the relationship between exercise intention and behavior and provide a basis for formulating personalized intervention strategies for individuals in different contexts.

### The mediating mechanism between exercise intention and behavior

As research deepens, scholars have begun incorporating variables to bridge the intention-behavior gap in exercise, such as planning (Gao et al., 2024), action control (Liu X. M. et al., 2022), exercise commitment (Zhang et al., 2022), self-identity (Chen et al., 2022), and positive wellbeing (Xu Z. et al., 2018), all of which have been shown to mediate the relationship between exercise intention and behavior. However, due to the limited number of original studies, this research focuses solely on the examination and validation of the roles of planning and action control.

The plan can be divided into two aspects: action planning and coping planning. Action planning is an extension of intention execution, encompassing specific situational parameters (such as time, place, etc.) and a series of actions (i.e., how to implement the plan). It effectively predicts the likelihood of behavior occurring (Gao et al., 2012). Coping planning refers to anticipating potential obstacles during the implementation of the intention and formulating strategies to overcome them (Caudroit et al., 2014). In their studies, Conner, Scholz, and Sniehotta introduced planning as a mediating variable, which increased the explanatory power of exercise behavior by 4%, 6%, and 13%, respectively (Conner et al., 2010; Scholz et al., 2008; Sniehotta et al., 2005a), while Norman et al. observed only a 2% increase in explanatory power after introducing planning (Norman and Conner, 2005). The inconsistency between these studies reveals a complex issue, namely that the mediating effect of planning is not fixed and may be influenced by other factors. This suggests that, although planning is an effective self-regulation strategy to facilitate behavior change, its actual effectiveness still requires a comprehensive evaluation considering individual differences and environmental factors. To fully understand the mediating effect of planning, it is essential not to focus solely on a single variable but to explore the factors that influence the efficiency of plan implementation, such as action control.

Action control, which refers to the integration of self-regulation processes such as goal awareness, self-monitoring, and effort (Sniehotta et al., 2005a), is a self-regulation strategy that coexists with planning and serves as a predictor more closely aligned with behavior. It facilitates the translation of exercise intentions into actual behavior (Liu X. M. et al., 2022). Notably, research indicates a sequential influence among exercise intentions, planning, action control, and exercise behavior. The explanatory power of variance in exercise behavior gradually increases with the addition of planning and action control to the model (18%−22%−26%; 11%−24%−32%; Sniehotta et al., 2005a; Liu X. M. et al., 2022). This suggests that action control not only independently influences behavior but may also indirectly affect outcomes by enhancing the execution efficiency of planning. However, current studies predominantly focus on the independent effects of action plans, coping plans, and action control on the transformation from exercise intention to behavior (Lee et al., 2024; Lee and Lee, 2020; Monge-Rojas et al., 2021; Pomp et al., 2010), limiting the depth of understanding regarding the relationship between intention and behavior. Furthermore, previous meta-analyses have yet to explore the independent or sequential mediating effects of planning and action control between exercise intention and behavior (Rhodes, 2024). Therefore, it is imperative to integrate past studies through meta-analytic structural equation modeling to test the relationships among variables and construct a model that validates the independent and sequential mediating roles of planning and action control from a broader perspective, and excluding the influence of certain confounding factors.

## Methods

### Literature search and selection criteria

The Chinese search terms included “锻炼意向,” “锻炼意图,” “运动意向,” “运动意图,” “锻炼行为,” “体力活动,” “体育锻炼,” “锻炼参与,” “计划,” “行动计划,” “应对计划,” “行动控制, “自我效能,” “自我效能,” and “习惯,” along with theories such as the 计划行为理论, 健康行动过程取向理论, 社会认知理论, and 双过程理论, which were used to search within the CNKI and Wanfang databases. For the English search, keywords such as “exercise intention,” “motion intention,” “motor intention,” “behavior intention,” “exercise behavior,” “exercise,” “physical activity,” “physical exercise,” “exercise participation,” “plan,” “action plan,” “coping plan,” “action control,” “self-efficacy,” “habit strength,” “habit,” and “habits,” along with theories including the TPB, HAPA theory, Social Cognitive Theory (SCT), and Dual Processes Theory (DPT), were utilized to conduct searches in databases such as Web of Science, ScienceDirect, Scopus, ProQuest, and EBSCO, with supplementary literature added through backward citation tracking. Given the timeliness of the research, the search was restricted to literature published from January 2001 to March 2024, yielding an initial collection of 1,622 articles.

**Figure 1:**
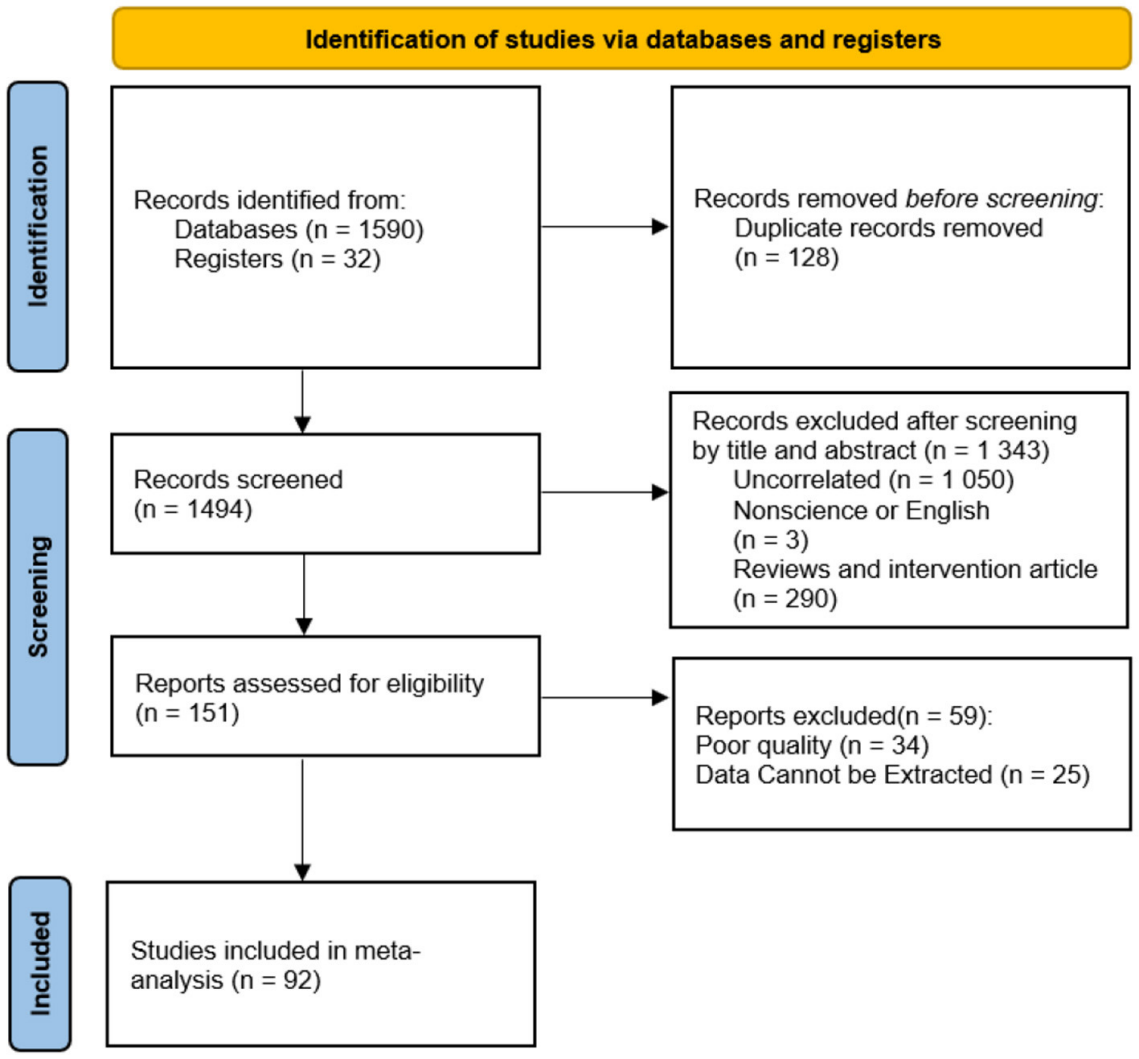
Flow diagram of literature screening and inclusion.

Selection Criteria: (1) Literature in both Chinese and English; (2) Type of literature: Quantitative empirical studies; (3) Research objective: To explore the relationship between exercise intention and behavior; (4) Research findings must clearly present sample size and effect size indicators. The literature selection was carried out collaboratively by two researchers, with disputed cases being resolved by a third researcher. A total of 92 articles were included, and the screening process is illustrated in Figure 1.

### Evaluation of literature quality and feature encoding

The literature was assessed for quality based on the 11 criteria recommended by the Agency for Healthcare Research and Quality (AHRQ). Responses were given as “Yes,” “No,” or “Unclear,” with “Yes” assigned 1 point, and “No” or “Unclear” receiving 0 points. The total score, with higher values indicating better quality, was classified as follows: a score of ≥8 indicated high quality, 6–7 indicated moderate quality, and ≤ 5 indicated low quality (Yang et al., 2019).

This study encodes data based on independent samples, with multiple codings conducted for studies involving several independent samples. The encoded data includes author information, publication year, sample size, age, gender, economic status, and cultural background, among others. The coding criteria are as follows: participants are categorized by age into adolescents (under 20), middle-aged adults (20–60 years), and elderly adults (over 60); economic status is classified according to World Bank standards into developed and developing countries; cultural background is divided into Eastern and Western cultures; socioeconomic status is determined by annual income, categorized as low (under $20,000), middle (between $20,000 and $100,000), and high (over $100,000); for studies involving students, educational stages are encoded, with only one study reporting primary school, which was therefore combined with secondary education (middle and high school); due to the lack of gender control in most studies, those with over 75% male or female participants are classified as predominantly male or female, respectively. The characteristics of the literature are presented in Table 1.

**Table 1:** Features of the literature included in the meta-analysis.

**References**	**Sample size**	**Mean age**	**Economic level and cultural background**	***r***	**AHRQ score**
Arbour and Ginis (2004)	47	46.6	Canada/West/Developed	0.44	6
Barg et al. (2012)	175	51.97	America/West/Developed	0.12	8
Blanchard et al. (2002a)	81	59.59	Canada/West/Developed	0.48	9
Blanchard et al. (2002b)	83	61.75	Britain/West/Developed	0.55	7
Blanchard et al. (2002b)	46	68.13	Britain/West/Developed	0.6	7
Blanchard et al. (2003)	215	59.52	Canada/West/Developed	0.05	7
Blanchard et al. (2007)	170	19.44	America/West/Developed	0.42	8
Blanchard et al. (2007)	180	19	America/West/Developed	0.45	8
Blanchard et al. (2009)	76	62.64	Canada/West/Developed	0.1	8
Blanchard et al. (2009)	76	62.64	Canada/West/Developed	0.31	8
Boudreau and Godin (2007)	92	47.7	Canada/West/Developed	0.62	9
van Bree et al. (2013)	1,836	62.95	Netherlands/West/Developed	0.29	7
van Bree et al. (2013)	636	62.95	Netherlands/West/Developed	0.25	7
van Bree et al. (2013)	554	62.95	Netherlands/West/Developed	0.23	7
van Bree et al. (2013)	646	62.95	Netherlands/West/Developed	0.12	7
van Bree et al. (2015)	1,976	63.63	Netherlands/West/Developed	0.35	8
Brickell et al. (2006)	162	23.15	Canada/West/Developed	0.69	8
Budden and Sagarin (2007)	274	–	America/West/Developed	0.5	6
Caudroit et al. (2014)	157	38.68	France/West/Developed	0.23	7
Chen et al. (2022)	1,573	13.71	China/East/Developing	0.345	7
Chiu et al. (2011)	195	47.35	America/West/Developed	0.36	10
Conner et al. (2010)	1,366	20.5	Britain/West/Developed	0.7	6
de Bruijn et al. (2009)	186	28.89	Netherlands/West/Developed	0.56	7
de Bruijn et al. (2009)	186	28.89	Netherlands/West/Developed	0.53	7
de Bruijn and Rhodes (2011)	538	21.19	Netherlands/West/Developed	0.31	8
de Bruijn et al. (2012a)	551	21.4	Netherlands/West/Developed	0.53	7
de Bruijn et al. (2012b)	413	21.4	Netherlands/West/Developed	0.53	7
de Bruijn et al. (2014)	586	21.6	Netherlands/West/Developed	0.66	6
Downs and Hausenblas (2003)	89	29.96	America/West/Developed	0.67	8
Downs et al. (2006)	338	14.28	America/West/Developed	0.7	9
Downs et al. (2006)	339	14.28	America/West/Developed	0.68	9
Downs and Hausenblas (2007)	62	30.44	America/West/Developed	0.48	9
Feng et al. (2023)	115	–	China/East/Developing	0.487	7
Fleig et al. (2013)	232	24.88	Germany/West/Developed	0.61	7
Gardner and Hausenblas (2004)	83	35.94	America/West/Developed	0.14	7
Gellert et al. (2012)	289	65	Germany/West/Developed	0.23	9
Gerber et al. (2011)	210	17.43	Switzerland/West/Developed	0.44	6
Hagger et al. (2001)	431	13	Britain/West/Developed	0.25	5
Hagger et al. (2007)	432	13.96	Britain/West/Developed	0.738	7
Hagger et al. (2007)	268	15.04	Estonia/West/Developed	0.713	7
Hagger et al. (2007)	150	14.35	Greece/West/Developed	0.482	7
Hagger et al. (2007)	235	14.01	Hungary/West/Developed	0.455	7
Hagger et al. (2007)	133	13.32	Singapore/East/Developed	0.765	7
Hamilton et al. (2017)	226	13.5	Australia/East/Developed	0.2	7
Hashim et al. (2014)	320	10.46	Malaysia/East/Developing	0.16	8
Hausenblas and Symons Downs (2004)	104	29.98	America/West/Developed	0.43	7
Hou et al. (2022)	218	19.53	China/East/Developing	0.42	9
Hu et al. (2023)	252	43.12	China/East/Developing	0.036	10
Johnson et al. (2015)	110	46.07	America/West/Developed	0.27	9
Karvinen et al. (2007)	354	64.5	Canada/West/Developed	0.53	8
Karvinen et al. (2009)	397	70.2	Canada/West/Developed	0.41	9
Keats et al. (2007)	59	18	Canada/West/Developed	0.44	7
Lee and Lee (2020)	740	17	Korea/East/Developed	0.48	6
Lee et al. (2024)	367	20.99	Korea/East/Developed	0.307	8
Lippke et al. (2004)	509	45	Britain/West/Developed	0.16	7
Lowe et al. (2002)	365	43.44	Britain/West/Developed	0.34	6
Luszczynska et al. (2010)	534	13.8	China/East/Developing	0.41	7
Luszczynska et al. (2010)	620	16.46	Poland/West/Developed	0.35	7
Ma et al. (2022)	1,166	14.51	China/East/Developing	0.265	9
MacCann et al. (2015)	1,017	23.1	America/West/Developed	0.59	7
Maher and Conroy (2015)	188	20.4	–	0.33	7
Monge-Rojas et al. (2021)	203	15.39	Costa Rica/West/Developed	0.46	5
Norman and Conner (2005)	125	21.38	Britain/West/Developed	0.6	8
Norman and Conner (2005)	102	20.80	Britain/West/Developed	0.7	8
Pfeffer and Strobach (2020)	108	37.17	Germany/West/Developed	0.35	7
Pfeffer et al. (2020)	191	22.70	Germany/West/Developed	0.45	8
Pomp et al. (2010)	290	49	Germany/West/Developed	0.32	9
Prapavessis et al. (2005)	58	28.84	NZ/West/Developed	0.26	6
Renner et al. (2007)	673	32	Korea/East/Developed	−0.04	8
Rhodes et al. (2003)	300	19.87	Canada/West/Developed	0.6	8
Rhodes and Courneya (2005)	585	20.07	Canada/West/Developed	0.63	7
Rhodes and De Bruijn (2010)	158	21.98	Canada/West/Developed	0.48	7
Rhodes and De Bruijn (2010)	179	21.98	Canada/West/Developed	0.62	7
Rhodes et al. (2010)	153	22.17	Canada/West/Developed	0.44	7
Roberts et al. (2010)	72	16.92	NZ/West/Developed	0.31	8
Rhodes et al. (2012)	216	24.02	Canada/West/Developed	0.52	6
Saunders et al. (2004)	1,797	13.6	America/West/Developed	0.334	7
Scholz et al. (2008)	354	37	Germany/West/Developed	0.37	6
Schwarzer et al. (2007)	365	37.01	Germany/West/Developed	0.15	7
Schwarzer et al. (2008)	353	58.8	Germany/West/Developed	0.19	9
Schwarzer et al. (2008)	114	54.3	Poland/West/Developed	0.32	9
Schwarzer et al. (2008)	368	47.4	Germany/West/Developed	0.39	9
Sheeran and Abraham (2003)	185	–	Britain/West/Developed	0.67	7
Sniehotta et al. (2005a)	307	59	Germany/West/Developed	0.3	8
Sniehotta et al. (2005b)	352	58.5	Germany/West/Developed	0.26	8
Sniehotta et al. (2010)	103	63	Germany/West/Developed	0.08	7
Stanley et al. (2012)	350	40.29	Britain/West/Developed	0.25	6
Teixeira et al. (2022)	215	36.21	Portugal/West/Developed	0.298	6
Vo and Bogg (2015)	957	49.61	America/West/Developed	0.64	6
Wang and Zhang (2016)	488	13.91	China/East/Developing	0.14	7
Wang and Kang (2024)	596	19.03	China/East/Developing	0.548	8
Liu W. et al. (2022)	1,312	14	China/East/Developing	0.265	7
Wiedemann et al. (2009)	124	60.3	Germany/West/Developed	0.36	6
Zhang et al. (2022)	581	19.27	China/East/Developing	0.526	7
Zhu et al. (2022)	589	20.61	China/East/Developing	0.42	8
Ziegelmann and Lippke (2007)	368	47.4	Germany/West/Developed	0.25	7
Bao et al. (2012)	331	37.8	China/East/Developing	0.251	6
Cao (2013)	534	13.8	China/East/Developing	0.23	7
Cao and Jiang (2013)	706	15.36	China/East/Developing	0.45	8
Gao et al. (2024)	299	55.67	China/East/Developing	0.687	9
Han et al. (2020)	207	43.12	China/East/Developing	0.294	7
Kang and Wang (2016)	353	11.14	China/East/Developing	0.3	7
Liu X. M. et al. (2022)	1,092	–	China/East/Developing	0.418	8
Wang and Zheng (2020)	751	–	China/East/Developing	0.336	7
Xu H. Y. et al. (2018)	2,080	14.5	China/East/Developing	0.153	6
Xu Z. et al. (2018)	303	19.85	China/East/Developing	0.305	9
Yang et al. (2020)	160	18.7	China/East/Developing	0.58	8
Yin et al. (2018)	1,111	14.74	China/East/Developing	0.21	9
Zhang et al. (2021)	2,302	–	China/East/Developing	0.28	6

### Data processing

This study uses the *Pearson correlation coefficient* (*r*) as the effect size and employs CMA software for data transformation. To mitigate the impact of sample size disparities across studies and address issues such as the absence of directly reported *r* values in some studies, we first convert the statistics from each study into correlation coefficients using the following formulas (Rupinski and Dunlap, 1996): $r=\sqrt{\frac{t^{2}}{t^{2}+df}}$; $r=\sqrt{\frac{x^{2}}{x^{2}+N}}$; $r=\sqrt{\frac{F}{F+df_{e}}}$; *r* = β × 0.98+0.05(β≥0); *r* = β × 0.98(β < 0). Next, the correlation coefficients are transformed using *Fisher's Z* transformation with the formula: $Z=\frac{1}{2}\times \ln\left(\frac{1+r}{1-r}\right)$, and the mean of the *Z* values is converted back to a correlation coefficient using: $r=\frac{e^{2x}-1}{e^{2x}+1}$. The *variance* of *Z*, $V_{z}=\frac{1}{n-3}$, and the *standard error* of *Z*, $SE_{z}=\sqrt{V_{z}}$. At this stage, invalid data may still remain in the studies, necessitating further checks for outliers, heterogeneity, and publication bias.

We begin with outlier detection, following the recommendations of Viechtbauer and Cheung (2010) under a random-effects model, using *studentized deleted residuals* to identify outliers (residuals > 2.5 indicate an outlier). Subsequently, we assess heterogeneity using the *Q* statistic and *I*^2^. A significant *Q* value (*p* < 0.05) indicates substantial heterogeneity in effect sizes; and *I*^2^ above 75% suggests high heterogeneity among studies. Finally, publication bias is evaluated using a funnel plot, *fail-safe N*, and *Egger's* test. The funnel plot examines the symmetry of effect sizes around the overall effect size to assess the risk, while an intercept close to 0 and non-significant in *Egger's linear regression* indicates no publication bias. Subgroup analyses are conducted to explore potential moderating variables, and the mediation effect is tested using the two-stage structural equation model based on correlation coefficients proposed by Cheung and Chan (2005). This involves first obtaining a joint correlation matrix through multivariate meta-analysis and then inputting this matrix into a structural equation model to test the mediation model (Wu and Fu, 2024). Finally, the *R*^2^ statistic is supplemented using Amos software.

## Results

### Results of literature inclusion and quality assessment

A total of 92 studies were included, encompassing 109 independent samples with 47,548 participants. The average quality score of the studies was 7.4. Among them, 86 studies reported the mean age of participants, comprising 42,829 individuals, with a quality score of 7.44. All 92 studies reported the gender of participants; however, only 19 studies, with a total of 6,061 participants, were included in the subgroup analysis based on subjective classification, and the quality score was 7.66. Forty-eight studies reported the educational level of participants, including 30,880 individuals, with a quality score of 7.28. All 92 studies reported the health status of participants, comprising 47,548 participants, with a quality score of 7.4. Fourteen studies reported the socioeconomic status of participants, with 6,013 individuals, and a quality score of 7.8. Ninety-one studies reported the cultural background and economic level of participants, totaling 47,360 individuals, with a quality score of 7.41. Overall, the quality of the included literature in this study was moderately high.

### Outlier detection

The examination of abnormal effect values and potentially risky effect sizes revealed that the residuals of 109 independent studies were all below 2.5, indicating the absence of outliers or effect sizes with potential risks, all of which were deemed suitable for inclusion in the meta-analysis.

### Publication bias examination

**Figure 2:**
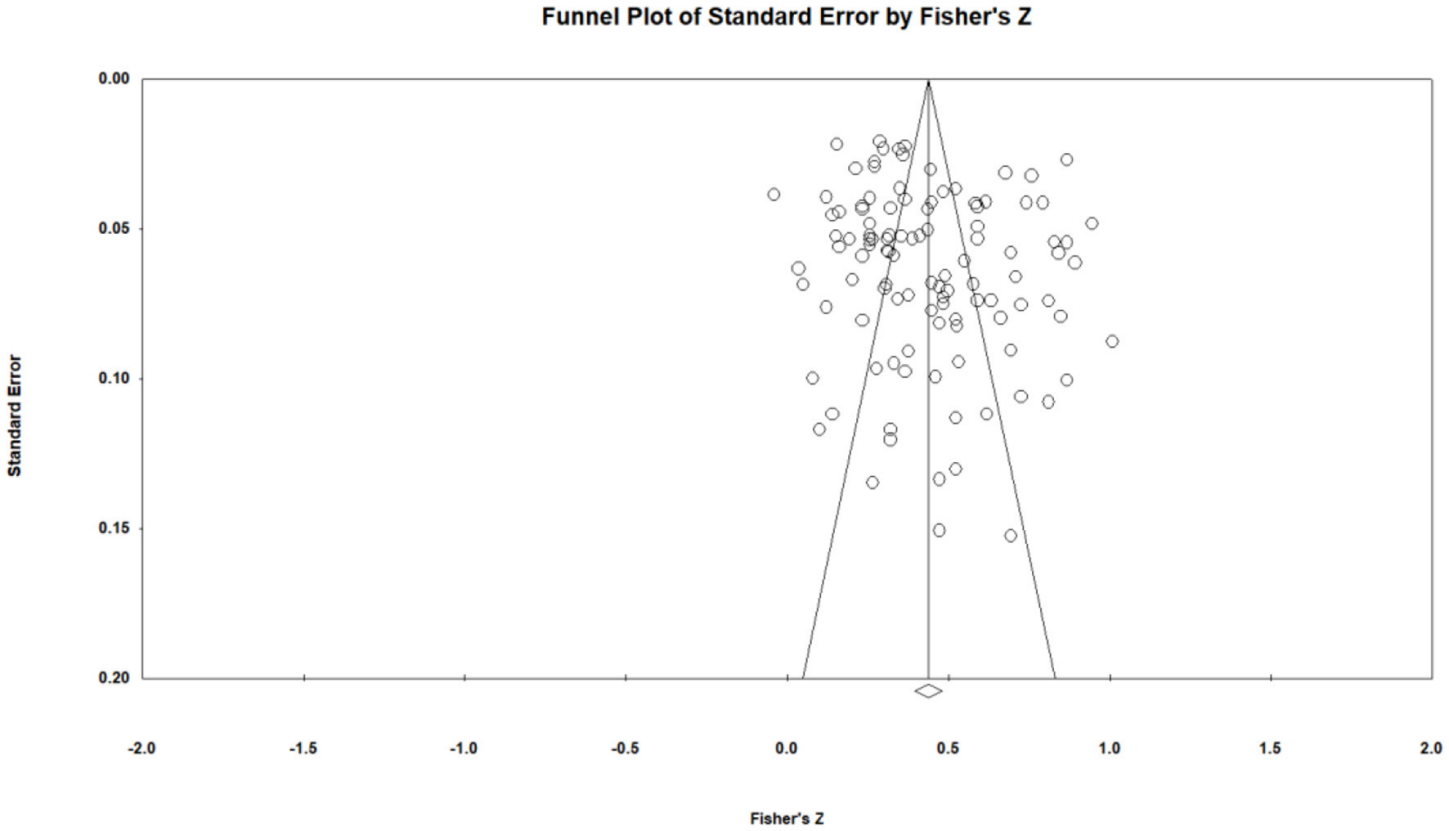
Funnel diagram of the relationship between exercise intention and exercise behavior.

The funnel plot's *x*-axis represents the effect size *Fisher's Z*, while the *y*-axis indicates the standard error of the *Z* value. The two diagonal lines demarcate the 95% confidence interval (Figure 2). The majority of the effect sizes are clustered toward the upper section of the funnel, with a nearly uniform distribution on both sides of the overall effect size. The fail-safe number is 95,783, exceeding the critical threshold of 555 (5*K*+10). The Egger's regression intercept is 1.84, with the intercept near zero and *p* > 0.05. Therefore, no significant publication bias exists in this study.

### Strength of the relationship between exercise intention and behavior

The meta-analysis reveals (Table 2) that the *Q*_*W*_ between exercise intention and exercise behavior is 2,235.6 (*p* < 0.001), with *I*^2^ = 95.17%, indicating significant heterogeneity among the studies. Therefore, a random-effects model was employed for analysis, along with a moderator effect test. The results show that the strength of the relationship between exercise intention and exercise behavior is *r* = 0.41 (*p* < 0.001). In other words, the actual intervention should focus on self-regulation strategies and external environmental factors to promote the transformation of exercise intention into behavior.

**Table 2:** Meta-analysis results of exercise intention, exercise behavior, action plan, coping plan, and action control.

**Variable**	***k***	***N***	***r***	**95% CI**	***I* ^2^**	**Heterogeneity**		
						***Q**_*W*_*	***df***	***p***
Exercise intention-exercise behavior	109	47,548	0.413	0.377/0.447	95.169	2235.601	108	< 0.001
Exercise intention-action plan	27	9,184	0.477	0.427/0.524	88.490	225.889	26	< 0.001
Exercise intention-coping plan	10	3,600	0.406	0.297/0.505	92.468	119.492	9	< 0.001
Exercise intention-action control	6	2,999	0.511	0.385/0.619	94.352	88.533	5	< 0.001
Exercise behavior-action plan	27	9,184	0.360	0.319/0.399	78.390	120.316	26	< 0.001
Exercise behavior-coping plan	10	3,600	0.343	0.274/0.408	78.694	42.242	9	< 0.001
Exercise behavior-action control	6	2,999	0.458	0.458/0.367	88.094	41.994	5	< 0.001
Action plan-coping plan	9	3,066	0.655	0.561/0.732	93.728	127.561	8	< 0.001
Action plan-action control	4	1,969	0.662	0.592/0.722	81.835	16.515	3	0.001
Coping plan-action control	1	1,092	0.700	0.668/0.729	–	–	–	–

k, number of effect values; N, sample size; r, correlation coefficient; 95% CI, 95% confidence interval for the overall correlation coefficient; I^2^, one of the heterogeneity test results; Q_W_, a statistic representing an intra-group heterogeneity test; df, degree of freedom.

### Examination of the moderating effects between exercise intention and behavior

This study tests the moderating effects based on participant characteristics, such as age, gender, and educational stage, to explore how the strength of the relationship between exercise intention and behavior is influenced by potential moderating factors, as shown in Table 3. Since all indicators are categorical variables, subgroup analysis was employed.

**Table 3:** Meta-analysis of moderating effects of subject characteristics.

**Moderation variable**	***k***	***N***	***r***	**95% CI**		***I* ^2^**	**Heterogeneity**		
							***Q**_*W*_*	***df***	***Q**_*B*_*
**Crowd**									
Teenager	33	18,420	0.43^***^	0.37	0.48	95.75	753.17^***^	32	8.27^*^
Middle age	56	17,213	0.42^***^	0.36	0.48	95.19	1143.42^***^	55	
Old people	14	7,196	0.31^***^	0.25	0.38	85.97	92.69^***^	13	
**Gender**									
Male	8	1,806	0.36^***^	0.18	0.51	93.61	109.58^***^	7	0.52
Female	13	4,255	0.43^***^	0.32	0.52	92.47	159.26^***^	12	
**Period of study**									
Primary and secondary	29	20,516	0.41^***^	0.35	0.46	95.95	691.96^***^	28	9.39^**^
Collegiate	28	10,364	0.53^***^	0.48	0.57	90.94	298.03^***^	27	
**Health status**									
Subhealth	30	5,774	0.43^***^	0.39	0.47	87.8	237.6^***^	29	4.09^*^
Health	79	41,774	0.35^***^	0.28	0.42	96.05	1974.81^***^	78	
**Socioeconomic status**									
Low	2	2,190	0.18^***^	0.08	0.27	34.68	1.53	1	10.15^**^
Middle	18	3,823	0.46^***^	0.32	0.58	96.26	455.04^***^	17	
**Culture**									
East	30	20,112	0.35^***^	0.3	0.41	94.86	564.22^***^	29	5.82^*^
West	78	27,248	0.44^***^	0.39	0.48	94.64	1437.5^***^	77	
**Economic level**									
Developed	83	29,387	0.43^***^	0.39	0.48	95.09	1669.83^***^	82	6.01^*^
Developing	25	17,973	0.35^***^	0.29	0.4	93.72	381.98^***^	24	

k, number of effect values; N, sample size; r, correlation coefficient; 95% CI, 95% confidence interval for the overall correlation coefficient; I^2^, one of the heterogeneity test results; Q_W_, a statistic representing an intra-group heterogeneity test; df, degree of freedom; Q_B_, a test statistic for inter-group heterogeneity; ^*^p < 0.05, ^**^p < 0.01, ^***^p < 0.001.

Subgroup analysis by age reveals that the average effect size for adolescents was 0.43 (95% CI = [0.37, 0.48]), *Z* = 12.33, *p* < 0.001; for middle-aged individuals, the average effect size was 0.42 (95% CI = [0.36, 0.48]), *Z* = 12.59, *p* < 0.001; for older adults, the average effect size was 0.31 (95% CI = [0.23, 0.38]), *Z* = 8.9, *p* < 0.001. Between-group comparisons show significant differences in effect sizes across age groups (*Q*_*B*_ = 8.27, *df* = 2, *p* = 0.016), indicating that the relationship between exercise intention and behavior is influenced by age. In the behavioral intervention, the predictive power of exercise intention on behavior can reach the medium level in the young and middle-aged group, while the predictive power is weak in the old group. Subsequent tests for within-group heterogeneity across age groups indicate significant variability (adolescents: *Q*_*W*_ = 753.17, *df* = 32, *p* < 0.001, *I*^2^ = 95.75; middle-aged: *Q*_*W*_ = 1,143.42, *df* = 55, *p* < 0.001, *I*^2^ = 95.19; older adults: *Q*_*W*_ = 92.69, *df* = 13, *p* < 0.001, *I*^2^ = 85.97), suggesting that the relationship between exercise intention and behavior differs within each age group, influenced by other moderating variables.

A subgroup analysis by gender revealed that the average effect size for males was 0.36 (95% CI = [0.18, 0.51]), *Z* = 3.86, *p* < 0.001, while for females it was 0.43 (95% CI = [0.32, 0.52]), *Z* = 7.2, *p* < 0.001. A comparison between the groups showed no significant difference in the effect sizes between genders (*Q*_*B*_ = 0.52, *df* = 1, *p* = 0.473), indicating that the relationship between exercise intention and behavior is not influenced by gender. In practice, there is no need to develop differentiated intervention strategies based on gender. Further, an assessment of within-group heterogeneity for each gender revealed significant heterogeneity in both groups (Male: *Q*_*W*_ = 109.58, *df* = 7, *p* < 0.001, *I*^2^ = 93.61; Female: *Q*_*W*_ = 159.26, *df* = 12, *p* < 0.001, *I*^2^ = 92.47), suggesting that the relationship between exercise intention and behavior differs by gender and is influenced by other moderating variables.

A subgroup analysis by educational level revealed that the mean effect size for primary and secondary school students was 0.41 (95% CI = [0.35, 0.46]), *Z* = 12.05, *p* < 0.001, while for university students, it was 0.53 (95% CI = [0.48, 0.57]), *Z* = 17.23, *p* < 0.001. A comparison between groups indicated a significant difference in effect sizes across educational levels (*Q*_*B*_ = 9.39, *df* = 1, *p* = 0.002), suggesting that the relationship between exercise intention and behavior is influenced by the educational stage. In behavioral intervention, the predictive power of exercise intention on behavior can reach the medium level in college students, while the predictive power is weak in primary and middle school students. Further examination of within-group heterogeneity revealed significant variability within both groups (primary and secondary school students: *Q*_*W*_ = 691.96, *df* = 28, *p* < 0.001, *I*^2^ = 95.95; university students: *Q*_*W*_ = 298.03, *df* = 27, *p* < 0.001, *I*^2^ = 90.94), indicating that the relationship between exercise intention and behavior differs across educational levels, with other moderating variables exerting influence.

A subgroup analysis of health status was conducted, revealing that the mean effect size for the healthy population was 0.43 (95% CI = [0.39, 0.47]), *Z* = 18.34, *p* < 0.001, while the mean effect size for the subhealthy population was 0.35 (95% CI = [0.28, 0.42]), *Z* = 9.33, *p* < 0.001. Inter-group comparisons indicated a significant difference in effect sizes across health status groups (*Q*_*B*_ = 4.09, *df* = 1, *p* = 0.043), suggesting that the relationship between exercise intention and behavior is influenced by health status. In behavioral intervention, the predictive power of exercise intention on behavior can reach the medium level in healthy people, while the predictive power is weak in sub-healthy people. Subsequently, within-group heterogeneity tests were conducted for each health status group. The results revealed significant heterogeneity within the groups (healthy group: *Q*_*W*_ = 1,974.81, *df* = 78, *p* < 0.001, *I*^2^ = 96.05; subhealthy group: *Q*_*W*_ = 237.6, *df* = 29, *p* < 0.001, *I*^2^ = 87.8), indicating that the relationship between exercise intention and behavior varies across different health statuses and is influenced by other moderating variables.

A subgroup analysis of socioeconomic status revealed that the average effect size for low socioeconomic status was 0.18 (95% CI = [0.08, 0.27], *Z* = 3.62, *p* < 0.001), while for moderate socioeconomic status, it was 0.46 (95% CI = [0.32, 0.58], *Z* = 5.74, *p* < 0.001). Between-group comparisons showed a significant difference in effect sizes across socioeconomic status levels (*Q*_*B*_ = 10.15, *df* = 1, *p* = 0.001), indicating that the relationship between exercise intention and behavior is influenced by socioeconomic status. In the behavioral intervention, the predictive power of exercise intention on behavior was moderate in the middle socioeconomic status group, but weak in the low socioeconomic status group. Subsequently, within-group heterogeneity tests revealed significant heterogeneity (low socioeconomic status group: *Q*_*W*_ = 1.53, *df* = 1, *p* = 0.216, *I*^2^ = 34.68; moderate socioeconomic status group: *Q*_*W*_ = 455.04, *df* = 17, *p* < 0.001, *I*^2^ = 96.26), suggesting the relationship between exercise intention and behavior varies across different socioeconomic status, influenced by other moderating variables.

A subgroup analysis of cultural backgrounds was conducted, revealing that the average effect size for Eastern cultures was 0.35 (95% CI = [0.3, 0.41]), *Z* = 11.44, *p* < 0.001, while the average effect size for Western cultures was 0.44 (95% CI = [0.39, 0.48]), *Z* = 17.25, *p* < 0.001. A comparison between groups indicated a significant intergroup effect size difference (*Q*_*B*_ = 5.82, *df* = 1, *p* = 0.016), suggesting that the relationship between exercise intention and behavior is influenced by cultural background. In behavioral intervention, the predictive power of exercise intention to behavior is moderate in Western cultures, but weak in Eastern cultures. Subsequent tests for within-group heterogeneity showed significant results (Eastern culture group: *Q*_*W*_ = 564.22, *df* = 29, *p* < 0.001, *I*^2^ = 94.86; Western culture group: *Q*_*W*_ = 1,437.5, *df* = 77, *p* < 0.001, *I*^2^ = 94.64), indicating that the relationship between exercise intention and behavior differs across cultural contexts and is moderated by other variables.

A subgroup analysis of national economic levels was conducted, revealing that the mean effect size for developed countries was 0.43 (95% CI = [0.39, 0.48]), *Z* = 17.03, *p* < 0.001, while for developing countries, the mean effect size was 0.35 (95% CI = [0.29, 0.4]), *Z* = 11.76, *p* < 0.001. Inter-group comparisons indicated a significant difference in effect sizes between groups with varying economic levels (*Q*_*B*_ = 6.01, *df* = 1, *p* = 0.014), suggesting that the relationship between exercise intention and behavior is influenced by the national economic level. In behavioral intervention, the predictive power of exercise intention to behavior is moderate in developed countries, but weak in developing countries. Further within-group heterogeneity tests revealed significant heterogeneity (developed countries: *Q*_*W*_ = 1,669.83, *df* = 82, *p* < 0.001, *I*^2^ = 95.09; developing countries: *Q*_*W*_ = 381.98, *df* = 24, *p* < 0.001, *I*^2^ = 93.72), indicating that the relationship between exercise intention and behavior remains diverse across different economic contexts, influenced by other moderating variables.

### Examination of the mediating effects between exercise intention and behavior

Firstly, the joint correlation matrix was computed. A homogeneity test was conducted on the model of the relationship between exercise intentions and behavior, revealing a poor model fit, χ^2^/(*df* =199) = 14.126, *p* < 0.001, which violated the homogeneity assumption. Therefore, following the recommendation of Cheung and Cheung (2016), a random effects model was used to estimate the joint correlation matrix (Table 4).

**Table 4:** Joint correlation matrix.

**Variable**	**1**	**2**	**3**	**4**	**5**
Exercise intention	–				
Action plan	0.463^***^	–			
Coping plan	0.397^***^	0.641^***^	–		
Action control	0.487^***^	0.651^***^	0.694^***^	–	
Exercise behavior	0.394^***^	0.354^***^	0.345^***^	0.448^***^	–

^*^p < 0.05, ^**^p < 0.01, ^***^p < 0.001.

**Figure 3:**
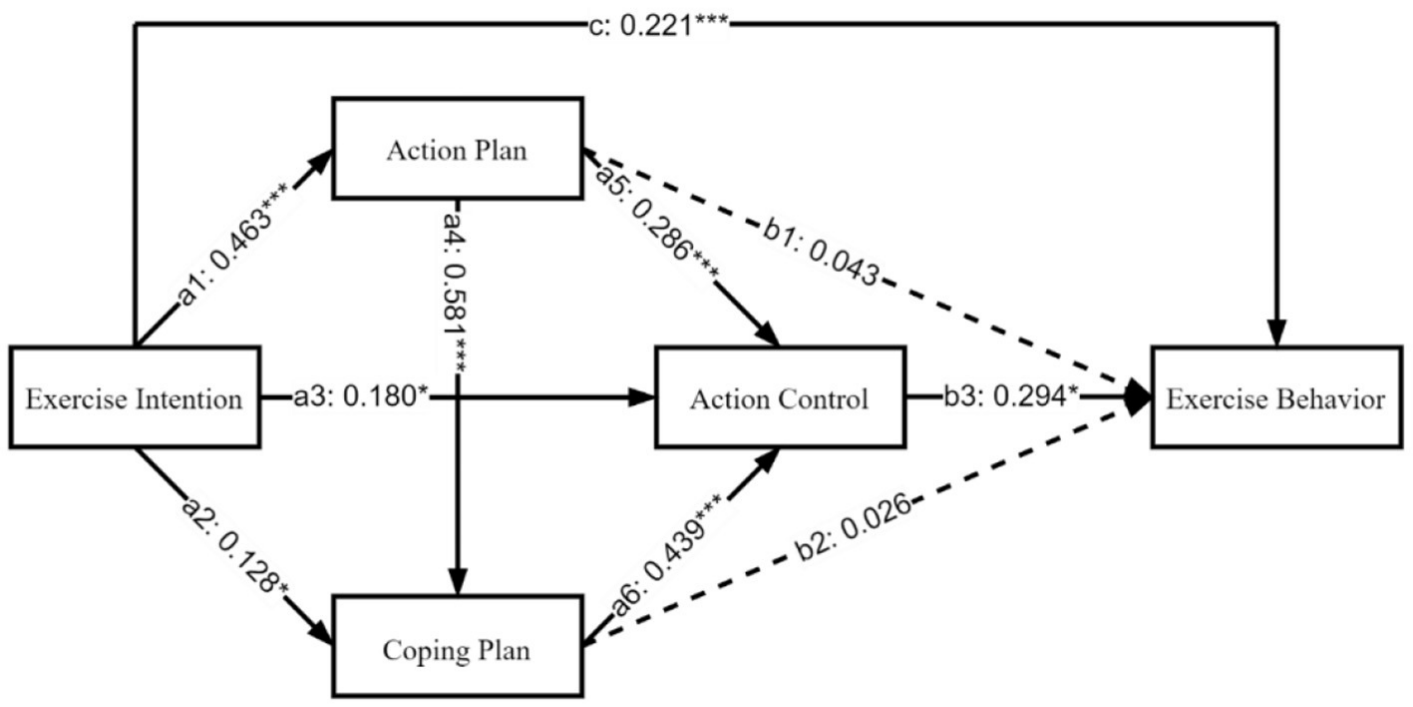
The role of planning and action control in the chain mediation between exercise intention and behavior. ^*^p < 0.05, ^**^p < 0.01, ^***^p < 0.001.

Next, path analysis was performed using the lavaan package (Jak et al., 2021), fitting the mediation model M1 as shown in Figure 3. The results indicated that, except for the paths from action plans and coping plans to exercise behavior, which were not significant, all other paths were significant, suggesting that exercise intentions primarily influenced exercise behavior through their direct effects and the singular and chain mediating roles of action plans, coping plans, and action control.

As shown in Table 5, exercise intention positively predicted action plans (*a*_1_ = 0.463), coping plans (*a*_2_ = 0.128), and action control (*a*_3_ = 0.180). Subsequently, these factors influenced exercise behavior (*b*_3_ = 0.294) through the chain mediation of action control (*a*_5_ = 0.286, *a*_6_ = 0.439) and single mediation. Thus, exercise intention indirectly influenced exercise behavior through the single mediation of action control (*a*_3_*b*_3_ = 0.053), through the chain mediation of action plans and action control (*a*_1_*a*_5_*b*_3_ = 0.039), through the chain mediation of coping plans and action control (*a*_2_*a*_6_*b*_3_ = 0.0.016), and through the chain mediation of action plans, coping plans, and action control (*a*_1_*a*_4_*a*_6_*b*_3_ = 0.035). Based on the total effect (*c*_t_ = 0.394), it was found that the single mediation effect of action control accounted for 13.45%, in other words, the path of direct conversion of exercise intention into behavior through action control is the most significant, indicating that immediate execution is the core driving force of behavior transformation; The chain mediation effect of action plans and action control accounted for 9.90%, that is, first through the preparation of action plan, and then with the help of action control implementation, reflecting the classic behavior change logic of “planning first”; The chain mediation effect of coping plans and action control accounted for 4.06%, that is, the plan for obstacles is implemented through action control, reflecting the value of “resilient response” to behavior maintenance; And the chain mediation effect of action plans, coping plans, and action control accounted for 8.88%, the complete chain from intention to plan and then to executive control shows that systematic planning can improve the efficiency of behavior transformation.

**Table 5:** Mediated effect path analysis.

**Path**	**Effect size**	**Path**	**Effect size**	**Path**	**Effect size**
*a*_1_	0.463 (0.416, 0.510)	*b*_1_	0.043 (−0.081, 0.150)	*a*_1_ *a*_4_ *b*_2_	0.007 (−0.039, 0.054)
*a*_2_	0.128 (0.019, 0.233)	*b*_2_	0.026 (−0.142, 0.190)	*a*_1_ *a*_5_ *b*_3_	0.039 (0.007, 0.086)
*a*_3_	0.180 (0.008, 0.349)	*b*_3_	0.294 (0.239, 0.519)	*a*_2_ *a*_6_ *b*_3_	0.016 (0.002, 0.042)
*a*_4_	0.581 (0.484, 0.680)	*a*_1_ *b*_1_	0.020 (−0.038, 0.069)	*a*_1_ *a*_4_ *a*_6_ *b*_3_	0.035 (0.007, 0.067)
*a*_5_	0.286 (0.136, 0.422)	*a*_2_ *b*_2_	0.003 (−0.024, 0.027)	*c*_ind_	0.173
*a*_6_	0.439 (0.349, 0.527)	*a*_3_ *b*_3_	0.053 (0.003, 0.107)	*c*_t_	0.394
*c*	0.221 (0.134, 0.287)				

a_1_, a_2_, a_3_ refers to the influence of exercise intention on action plan, coping plan and action control, respectively, a_4_, a_5_ refers to the influence of action plan on coping plan and action control respectively, a_6_ refers to the influence of coping plan on action control, c refers to direct influence of exercise intention on exercise behavior, b_1_, b_2_, b_3_ refers to the influence of action plan, coping plan and action control on exercise behavior, respectively. a_3_b_3_: exercise intention → action control → exercise behavior; a_1_a_5_b_3_: exercise intention → action plan → action control → exercise behavior; a_2_a_6_b_3_: exercise intention → coping plan → action control → exercise behavior; a_1_a_4_a_6_b_3_: exercise intention → action plan → coping plan → action control → exercise behavior; c_ind_: The total indirect influence of the predictor variable on the outcome variable through mediation; c_t_: The total influence of the predictor variable on the outcome variable, the same below.

Additionally, the model was constructed using Amos to supplement the variance explanation indicator. The results revealed that the zero model (direct effect) had an *R*^2^ = 0.28, while the full model (serial mediation effect) showed *R*^2^ = 0.37.

## Discussion

### The strength of the relationship between exercise intention and behavior

This study employs a meta-analysis to integrate 47,548 participants across 92 studies, revealing a significant positive correlation between exercise intention and behavior. In contrast to previous individual studies (Ma et al., 2022; Zhang et al., 2022), this meta-analysis, by synthesizing a substantial body of research, substantiates that exercise intention is a key predictor of exercise behavior. The direct effect model's *R*^2^ = 0.28, indicating that a considerable variance in exercise behavior remains unexplained by intention alone. This finding mirrors that of Hamilton et al. (2017), who found that while exercise intention predicts behavior, a substantial “intention-behavior gap” persists. This suggests that other variables may account for the discrepancy between intention and behavior. These variables could stem from the multifaceted, layered mechanisms underlying intention, including complex external factors (such as environment and cultural context) and internal factors (such as age, gender, academic level, and socioeconomic status) and their interactions.

### The moderating effects of participant characteristics

This study, through subgroup analysis, explores the moderating effects of demographic variables—such as age, educational level, health status, socioeconomic status, cultural background, and economic level—on the relationship between exercise intentions and behavior. This not only deepens our understanding of the link between exercise intentions and behavior but also provides valuable insights for the formulation of more precise behavior change strategies.

The moderating effects of age and educational level are significant, with the effect sizes for adolescents, middle-aged individuals, and college students being notably higher than those for the elderly and primary or secondary school students. Consistent with previous meta-analytic findings (Rhodes and Dickau, 2012), this may be attributed to the influence of leisure time and health status. Middle-aged adults and college students typically have stable work or study schedules, affording them fixed, discretionary leisure time. In contrast, adolescents in middle and high school are often constrained by academic pressures, making it difficult to translate exercise intentions into action (Zhang and Mao, 2016), while older adults are more restricted by health concerns. Similarly, the moderating effect of health status is significant, with healthier individuals showing higher effect sizes compared to those in suboptimal health. This may be due to the fact that individuals in suboptimal health, despite having strong exercise intentions, are often unable to engage in certain types of exercise due to physical limitations (Watson et al., 2023). Given the individual differences in the translation of exercise intentions into behavior, intervention strategies should focus on personalization, taking into account the specific needs and realities of different groups.

The moderating effect of socioeconomic status is significant, with individuals of middle socioeconomic status exhibiting notably higher effect sizes than those of lower status. This may be attributed to the fact that individuals with higher socioeconomic status typically have more time and financial resources, whereas those with lower socioeconomic status are often constrained by economic limitations and the pressures of balancing work and life (Marmot, 2005). The moderating effect of cultural background is also significant, with the effect size in Western cultures being markedly higher than in Eastern cultures. This can be explained by the greater emphasis on individualism in the West, while in Eastern collectivist cultures, the purpose of exercise is more inclined toward social interaction. For instance, Chinese university students often engage in sports clubs not only for physical health and skill acquisition, but also to strengthen social ties and a sense of belonging to the campus community. In such a cultural context, the transformation of exercise intention into behavior may rely more on group dynamics and social support than on individual decision-making. The moderating effect of national economic level is significant as well, with developed countries showing higher effect sizes than developing ones. This may be because economically developed countries tend to offer better quality sports facilities, higher per capita disposable income, better health awareness, and more comprehensive social welfare and healthcare systems, thus encouraging greater participation in physical exercise. Economic development not only influences the accessibility of fitness resources, but is also closely linked to public health awareness and lifestyle choices, highlighting that the formation of health consciousness and the availability of resources are both crucial issues to address.

However, the current meta-analysis found no moderating effect of gender on the relationship between exercise intention and behavior, consistent with previous meta-analytic results (Rhodes and Dickau, 2013). From the perspective of effect size, women exhibited a better conversion of exercise intention to behavior. This may be attributed to the greater likelihood of women receiving support and encouragement from friends, family, or colleagues during exercise, thereby enhancing the transition from intention to behavior. Moreover, the gender classification in this study was not dichotomous (0 and 1), but rather proportional, which may introduce some bias in the results. Future research could group participants by gender to design more targeted intervention strategies based on gender-specific characteristics.

It is worth noting that the heterogeneity within the selected moderating variables in this study remains considerable, indicating that there are still potential influencing factors yet to be uncovered. This raises the question of whether interactions might exist among the moderating variables. For example, in adolescent groups, the influence of school stage may still be a factor; for middle-aged individuals, socioeconomic status may play a significant role; and for the elderly, health status might be a key determinant. Therefore, future research is encouraged to adopt a more holistic perspective (such as exploring the interplay between individual exercise intentions, behaviors, and the environment based on social ecological theory) and more targeted statistical methods (such as utilizing multilevel linear models to explore the complex relationships and interactions among variables). This approach will help to gain a more comprehensive understanding of the relationship between exercise intentions and behaviors, as well as the mechanisms of their transformation.

### The mediating role of planning and action control

The meta-analysis results indicate that planning and action control serve as chain mediators in the mechanism by which exercise intention affects behavior. This undoubtedly offers inspirations for the formulation of intervention strategies. With strengthening action control as the core target (the single mediator with the highest proportion (13.45%) + the necessary link in all chain paths), supplemented by action plans (such as providing personalized exercise templates) and response plans (such as obstacle response workshops), the implementation of exercise behavior can be better facilitated. Secondly, precise intervention should be implemented hierarchically. For the primary group (those with weak executive ability), the single path of “action control” should be the main focus (low cost and easy to operate), such as sending daily text message reminders for exercise; for the advanced group (those with basic planning ability), the chain path of “action plan → action control” should be activated, such as pushing customized weekly plans and synchronizing them to the calendar; for the high-level group (those facing multiple obstacles), the complete chain path should be initiated, for example, providing a trinity intervention of “goal—obstacle response plan—execution tracking” for patients with chronic diseases. In practice, emphasize “control” to promote execution; in research, break through “static” to achieve precision; in technology, integrate “intelligence” to enhance efficiency.

Notably, in the chain mediation model, the direct effect of action plans and coping plans on exercise behavior is not significant, with their influence instead mediated through action control. This suggests that the impact of planning on exercise behavior may be mediated by action control, aligning with the findings of previous studies (Lee et al., 2024; Monge-Rojas et al., 2021; Liu X. M. et al., 2022). The reasons for this phenomenon may be as follows: First, individuals may encounter various obstacles both before and after implementing plans. In such circumstances, whether an individual can activate coping plans through action control, rather than choosing to abandon the plan, becomes crucial in overcoming action barriers. Second, once behavior is initiated, individuals need to continuously engage in self-monitoring and effort to close the gap between their behavior and the intended standard. Third, individuals who have already developed exercise habits may no longer require plans. Furthermore, self-efficacy can moderate the relationship between exercise intention and behavior through planning (Di Maio et al., 2021; Lippke et al., 2009); increased habit strength reduces the need for strong exercise intention for the occurrence of exercise behavior (van Bree et al., 2013; Di Maio et al., 2021); positive emotions associated with past exercise can promote future participation in exercise (Zhang and Mao, 2016); and exercise identity can directly predict or indirectly influence exercise behavior through intention (Jackson et al., 2003). These variables may, to some extent, attenuate the mediating role of planning. A recent study published in the BMJ emphasized the importance of the effort-minimization theory (i.e., the natural human tendency to reduce physical activity participation) in bridging the intention-behavior gap, and pointed out that executive functions and emotional experiences may be key to overcoming the automatic pull of effort minimization, with this tendency being influenced by individual differences and situational factors (Cheval et al., 2024). However, there is still a lack of high-quality evidence supporting this theory, with few studies conducted, and its applicability across different populations has not yet been explored. Therefore, future research is recommended to delve into how individual differences and environmental factors influence the relationship and interactions between exercise intention and behavior. Additionally, a mixed research approach combining qualitative research (to uncover specific barriers and success experiences individuals encounter in the behavior change process) and quantitative research should be employed to comprehensively understand the complexity of behavior change.

### Limitations and future research

**Figure 4:**
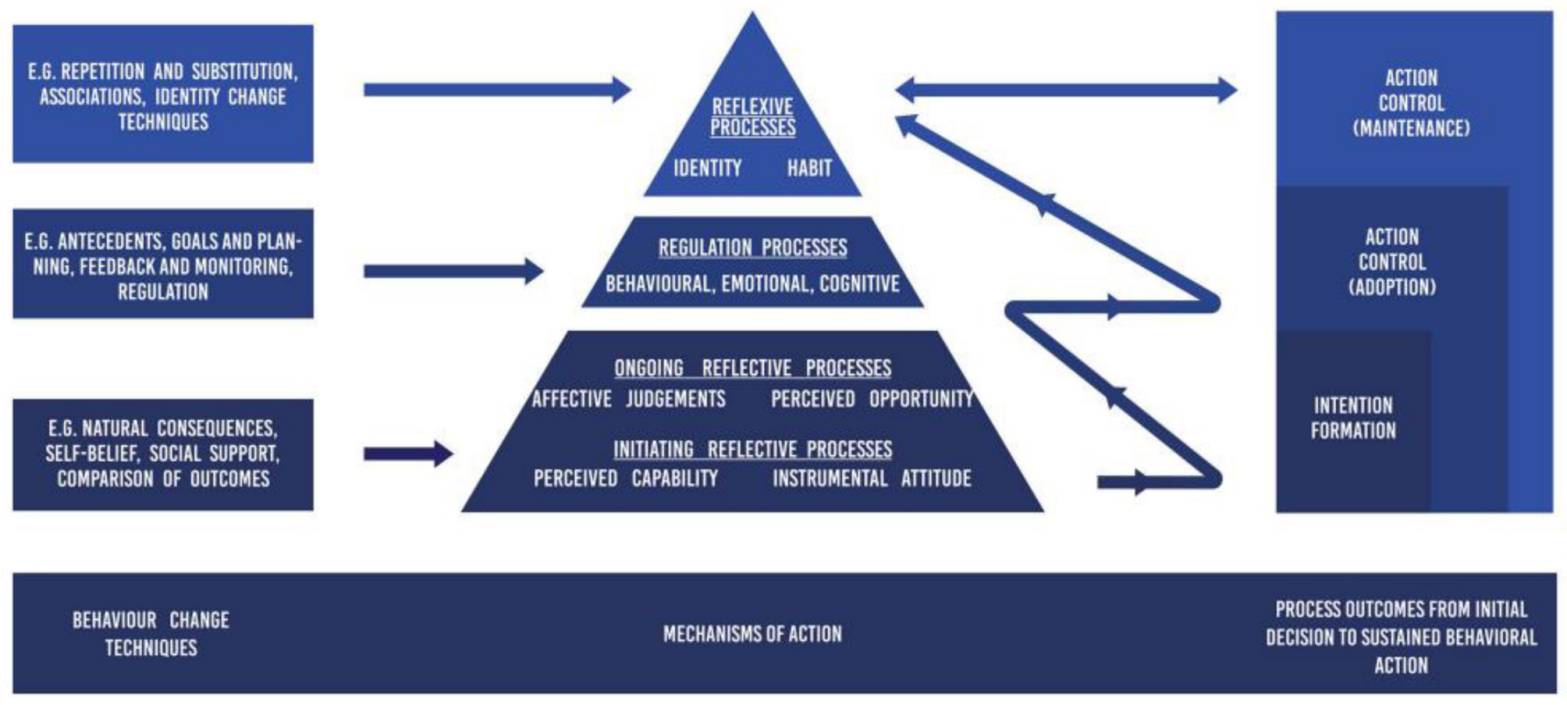
Schematic diagram of multi-process action control framework (Rhodes, 2021).

Research indicates that the proportions of successful and unsuccessful non-intenders are 4.2% and 26.0%, respectively, while for the successful and unsuccessful intenders, the proportions are 33.0% and 38.7% (Feil et al., 2023). This suggests that a significant part of the stability in the relationship between exercise intention and behavior comes from those who neither intend nor engage in exercise (26%). However, few individuals report being non-intenders, which could lead to a misunderstanding of the connection between exercise intention and behavior. To better address such issues, models based on action control processes that bridge the intention-behavior gap have been gaining popularity, particularly with the integration of various exercise behavior theories. For instance, the Multi-Process Action Control (M-PAC) framework, which originated from traditional social cognitive models and has been integrated and refined (Rhodes, 2017). This framework posits that exercise behavior is influenced by three broad hierarchical factors: reflective processes, regulatory processes, and reflective processes (Figure 4). First, the reflective process represents the conscious and deliberate anticipated outcomes of engaging in physical activity (i.e., intention formation); the regulatory process involves the behavioral or cognitive regulation by which individuals act to translate their intentions into exercise behavior; the reflective process reflects automatic influences on action control, such as habits and identity. Furthermore, the M-PAC framework has a causal structure, from intention formation to behavior initiation and maintenance, yet each process interacts and reinforces the others. Therefore, while the M-PAC framework represents an orderly acquisition of reflective, regulatory, and reflective processes, these processes evolve over time and interact, with each expected to make an independent contribution or act as a mediator in the intention-behavior transformation. For instance, for non-intenders, the primary issue is to stimulate their exercise intention (motivation formation); thus, can motivation theories such as self-determination theory, along with the reflective process in the M-PAC framework, more effectively promote the formation of exercise intention and behavior change among non-intenders? The challenge for intenders lies in actively engaging in behavior and overcoming barriers to action. Therefore, do theories such as health action process orientation and the regulatory process in the M-PAC framework apply more effectively in the process of exercise behavior change? After behavior initiation, the reflective process in the M-PAC framework—such as exercise habits and exercise identity—could have a stronger effect on behavior maintenance. These issues remain underexplored and lack high-quality research for validation. Hence, future studies should enhance the application of the M-PAC framework in the field of exercise behavior. Specifically, on one hand, it is essential to distinguish between intenders, non-intenders, behavior initiators (those who intend but have not engaged in exercise), and behavior maintainers (those who intend and have engaged in exercise), exploring the processes and factors influencing the successful transition of non-intenders into active participants, as well as the differences and reasons for behavior change among intenders. On the other hand, it should be examined whether the regulatory and reflective processes play decisive roles in the initiation and maintenance of behavior. This would not only reduce the influence of external factors but also contribute to the development of personalized exercise promotion strategies for different population groups.

Furthermore, current research predominantly utilizes cross-sectional methodologies, with few studies incorporating longitudinal tracking or randomized controlled trials. However, behavior change is a continuous process, and cross-sectional surveys or short-term tracking fail to effectively capture the dynamic fluctuations and causal relationships between exercise intentions and behaviors. Additionally, some interventions are limited to short-term outcomes and struggle to maintain long-term effects. Therefore, future research should focus on the dynamic process of transforming exercise intentions into behaviors. On one hand, intensive tracking methods should be employed to explore the internal psychological processes and dynamic changes within individuals over a short period, or long-term tracking (over 1 year) should be conducted to examine changes in exercise intentions and behaviors at different time points, along with their underlying causes (such as the formation of exercise habits and the emotions and feelings experienced during exercise). At the same time, exploration of other unknown variables (e.g., self-schemas) should be strengthened, or alternative research methods (e.g., latent growth models) should be utilized to investigate the trajectories of exercise intention and behavior changes, as well as the relationship between the two. On the other hand, intervention experiments are also essential, particularly those focusing on habit formation and personality trait differences among individuals with no initial intention to exercise, as nearly no one is born with a habit of engaging in physical exercise, and individuals with differing personality traits perceive the same events in distinct ways, potentially influencing behavior change.

Finally, current research predominantly relies on self-reported data collection methods. However, such data is susceptible to biases such as recall distortion and social desirability, which may compromise its accuracy. With the rapid advancement of wearable technology, future studies could employ devices such as wristbands or accelerometers to measure physical activity, thereby enhancing the reliability of data sources.

## Conclusions

The relationship between exercise intention and behavior exhibits a moderate strength (*r* = 0.41). In this relationship, with the exception of gender, age, educational stage, health status, socio-economic status, cultural background, and economic level all significantly moderate the connection between intention and behavior. Action plans, coping strategies, and action control serve as chain mediators between exercise intention and behavior, with action control (Single mediation effect was 13.45%) emerging as the closest predictor of actual behavior. Future research is recommended to rigorously control for participant characteristics, conduct long-term longitudinal studies, and implement intervention-based behavioral change experiments. Additionally, the application of objective measurement tools should be strengthened, and the exploration and analysis of new theories and variables should be deepened, to facilitate the transformation of exercise intention into behavior.

## Data Availability

The raw data supporting the conclusions of this article will be made available by the authors, without undue reservation.
